# Hemophagocytic Lymphohistiocytosis in a Patient With Advanced HIV and Cytomegalovirus Infection

**DOI:** 10.1177/2324709620906961

**Published:** 2020-02-13

**Authors:** Amir Anabtawi, Reem Alkilany, Mary E. Lacy

**Affiliations:** 1 University of New Mexico, Albuquerque, NM, USA; 2 MetroHealth Medical Center, Cleveland, OH, USA

**Keywords:** hemophagocytic lymphohistiocytosis, human immunodeficiency virus, cytomegalovirus, hyperferritinemia

## Abstract

Hemophagocytic lymphohistiocytosis (HLH) is a rare, aggressive, and, if not treated, fatal disorder that is characterized by excessive immune system activation. This disorder can be precipitated by different triggers including malignancies, infections, and autoimmune disorders. Diagnosis is made by fulfilling criteria that was last updated in 2004, and treatment frequently includes management of the underlying trigger but can also include chemotherapy. In this article, we report a case of HLH in a 27-year-old male, who had been diagnosed with advanced untreated HIV, who presented to the hospital with fever and generalized fatigue with no obvious etiology. Infectious workup revealed cytomegalovirus viremia, and the patient met HLH criteria with impressive hyperferritinemia of 15 432 ng/mL. The patient was started on treatment for cytomegalovirus infection that led to resolution of HLH. Our report highlights the importance of early detection of HLH in special populations, and that treating the presumptive trigger can lead to resolution of HLH.

## Introduction

Hemophagocytic lymphohistiocytosis (HLH) is a serious, aggressive, and life-threatening syndrome caused by a dysfunction of the immune system and is characterized by excessive immune system activation. It is most common in children but can affect patients of any age. Most patients have either a predisposing genetic defect and/or immunologic trigger such as infection, cancer, or rheumatologic disorder. We present a patient diagnosed with HLH due to cytomegalovirus (CMV) in the setting of newly diagnosed, advanced human immunodeficiency virus (HIV). In this case, the treatment of CMV infection resulted in resolution of HLH.

## Case Report

A 27-year-old man with a past medical history of alcohol and marijuana use disorders presented to an outside facility with fevers, fatigue, and night sweats. He was diagnosed there with advanced HIV and was found to have a CD4 count of 3/µL. The outside facility initiated an extensive workup that included computed tomography scan showing cavitary lung lesions, followed by bronchoscopy with bronchial biopsies that were all negative for acid-fast bacilli and *Pneumocystis jirovecii*. He received treatment for community-acquired pneumonia without any notable improvement and then was treated for presumed *Pneumocystis jirovecii* with atovaquone and steroids, again without notable improvement. A lumbar puncture was done that was negative for infectious or inflammatory conditions. The remainder of his workup at the outside hospital is shown in Table 1.

**Table 1.** Laboratory Values at Outside Facility and at Our Hospital.

Name of Test	Reference Range	Outside Hospital	Our Facility	Our Facility on Discharge (3 Weeks From Admission)
WBC, ×10^3^/µL	4-11	3.0	5.1	8.9
Hgb, g/dL	13.5-17.7	9.6	8.8	9.9
MCV	81-101		98	
Plt, ×10^3^/µL	150-400	49	45	125
INR	0.8-1.3		1.09	1.04
APTT, seconds	26-38		56	41
Fibrinogen, mg/dL	170-450		150	95
Ferritin, ng/mL	26-388		15 432	7930
AST, U/L	6-58	240	233	104
ALT, U/L	14-67	164	164	174
Alk phos, U/L	38-150	251	299	161
Triglyceride, mg/dL	<150		231	
LDH, U/L	117-224		655	
T bilirubin, mg/dL	0.3-1.2	0.6	0.6	0.9
D-dimer, ng/mL	0-500		6495	
Toxo IgG	<3	<3		<3
HCV Ab	Nonreactive	Negative		Negative
HBsAb	<10	Negative		Negative
HBsAg	Nonreactive	Negative		Negative
CD4, count/µL	500-1,500	3		
HIV viral load		156 000		
Cocci serum IgG		Negative		
Cocci serum IgM		Negative		
Crypto Ag, blood		Negative		
Crypto Ag, CSF		Negative		
Blood culture		Negative ×4		
AFB, CSF		Negative		
CMV, blood, copies/mL			10 million	

Abbreviations: WBC, white blood cell; Hgb, hemoglobin; MCV, mean corpuscular volume; Plt, platelets; INR, international normalized ratio; APTT, activated partial thromboplastin time; AST, aspartate aminotransferase; ALT, alanine aminotransferase; Alk phos, alkaline phosphatase; LDH, lactate dehydrogenase; T bilirubin, total bilirubin; Toxo, toxoplasmosis; HCV Ab, hepatitis C virus antibody; HBsAb, hepatitis B surface antibody; HBsAg, hepatitis B surface antigen; HIV, human immune deficiency virus; Cocci, coccidioidomycosis; Ig, immunoglobulin; Crypto, *Cryptococcus*; AFB, acid-fast bacilli; CSF, cerebrospinal fluid; CMV, cytomegalovirus.

He was ultimately transferred to our hospital given unexplained persistent fever, fatigue, and night sweats. He additionally reported abdominal pain and poor appetite. He denied nausea, vomiting, diarrhea, or constipation. He denied any personal or family history of recurrent infections, blood disorders, malignancy, or autoimmune disease.

**Figure 1.**
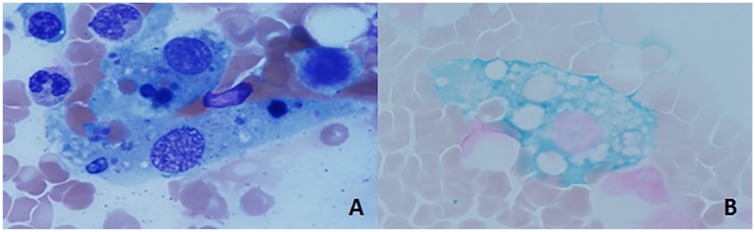
Bone marrow biopsy. (A) Iron-stained histiocyte/macrophage. (B) Hematoxylin and eosin stain of bone marrow biopsy demonstrating macrophage ingesting other cells.

On presentation to our facility, vital signs were significant for a temperature of 38.1°C and a heart rate of 102 beats per minute. His physical examination was notable for cachexia, oral thrush, mild hepatomegaly, and muscular weakness. Notably, the patient had a markedly elevated ferritin of 15 432 ng/mL, and shortly after presentation, the patient was found to have CMV viremia with more than 10 million copies/mL. Mild hepatic steatosis without splenomegaly was noted on ultrasound of the abdomen. Additional laboratory testing performed at our institution is shown in Table 1. There was concern for secondary HLH and a bone marrow biopsy showed hypocellular bone marrow with hemophagocytic activity (Figure 1). Bone marrow flow cytometry was consistent with active HIV infection.

Our patient met HLH-2004 criteria (Table 2), which includes fever, cytopenias, hypertriglyceridemia, hypofibrinogenemia, elevated serum transaminases, and a bone marrow biopsy demonstrating hemophagocytosis (Figure 1). Our patient’s ferritin level was >15 000 as well, which while not pathognomonic for HLH, has a narrow differential diagnosis that includes HLH. He was diagnosed with acute HLH secondary to CMV viremia in the setting of newly diagnosed advanced HIV.

**Table 2.** Our Patient’s Initial Clinical Presentation Met 5/8 HLH-2004 Diagnostic Criteria.

HLH Criteria	Our Patient
Fever ≥38.5°C	Present
Splenomegaly	Not present
Peripheral blood cytopenia, with at least 2 of the following: hemoglobin <9 g/dL; platelets <100 000/µL; absolute neutrophil count <1000/µL	Present
Hemophagocytosis in bone marrow, spleen, lymph node, or liver	Present
Low or absent NK cell activity	Not checked
Ferritin >500 ng/mL	Present
Elevated soluble CD25 (soluble IL-2 receptor-α) 2 standard deviations above age-adjusted laboratory-specific norms	Not checked
Hypertriglyceridemia (fasting triglycerides >265 mg/dL) and/or hypofibrinogenemia (fibrinogen <150 mg/dL)	Present

Abbreviations: NK: natural killer; HLH: hemophagocytic lymphohistiocytosis.

An interdisciplinary meeting, including infectious disease and hematology specialists, was held. We started the patient on valganciclovir 900 mg twice a day for the CMV, as well as steroid therapy with prednisone 40 mg daily. Following the initiation of treatment for CMV, our patient’s clinical status improved significantly with resolution of fevers, improvement in cytopenias, and a drastic reduction in the ferritin (Table 1).

## Discussion

HLH is a rare disease caused by a dysfunction of cytotoxic T-cells and natural killer cells. This T-cell/natural killer cell dysregulation causes an aberrant cytokine release, resulting in proliferation/activation of histiocytes with subsequent hemophagocytosis and ultimately excessive immune system activation. HLH was first described in 1939 by Scott and Robb-Smith.^1^ Understanding the epidemiology of HLH is a challenge given the rarity of the disease as well as the propensity of underdiagnosis and misdiagnosis. However, a nationwide survey done in Japan estimated the annual incidence of HLH to be 1 in 800 000 per year.^2^

HLH can be primary as a result of an underlying genetic defect in an array of genes such as PRF1, UNC13D, STX11, and other genes that is more commonly diagnosed in infants and children.^3^ The presentation of HLH is more often secondary, though, and triggered by malignancies (most commonly hematologic), autoimmune diseases (macrophage activation syndrome),^4^ or infections. Most reported infectious triggers are herpesviruses, most notably Epstein-Barr virus.^5^ A defect in granule-mediated cytotoxicity is the underlying common mechanism in both primary and secondary forms of HLH, leading to uncontrolled activation of antigen-presenting cells such as macrophages and histiocytes, and T-cells. This activation leads to hypersecretion of cytokines. This so-called cytokine storm could be pathogenically related to the development of the main clinical and laboratory features of HLH and contributes to tissue damage and progressive systemic organ failure.^6^ Treatment of HLH includes identifying and managing any underlying trigger, along with HLH-specific therapy based on HLH-94 protocol that includes cytotoxic plus immunosuppressive therapy such as etoposide and dexamethasone.^7^

A retrospective study recently evaluated HLH in patients with HIV. The study found CMV infection in 14% of the pooled patients; moreover, most of the reported patients had coinfection, typically with mycobacteria.^8^ To our knowledge, HLH caused by isolated CMV infection in the setting of HIV has rarely been reported. Of great importance in our patient, treatment of CMV infection led to resolution of HLH.
